# Examining the trade-offs between human fertility and longevity over three centuries using crowdsourced genealogy data

**DOI:** 10.1371/journal.pone.0255528

**Published:** 2021-08-05

**Authors:** Chen-Hao Hsu, Oliver Posegga, Kai Fischbach, Henriette Engelhardt

**Affiliations:** 1 Department of Sociology, University of Bamberg, Bamberg, Germany; 2 Department of Information Systems and Social Networks, University of Bamberg, Bamberg, Germany; 3 The State Institute for Family Research (ifb), Bamberg, Germany; University of Salamanca, SPAIN

## Abstract

The evolution theory of ageing predicts that reproduction comes with long-term costs of survival. However, empirical studies in human species report mixed findings of the relationship between fertility and longevity, which varies by populations, time periods, and individual characteristics. One explanation underscores that changes in survival conditions over historical periods can moderate the negative effect of human fertility on longevity. This study investigates the fertility-longevity relationship in Europe during a period of rapid modernisation (seventeenth to twentieth centuries) and emphasises the dynamics across generations. Using a crowdsourced genealogy dataset from the FamiLinx project, our sample consists of 81,924 women and 103,642 men born between 1601 and 1910 across 16 European countries. Results from multilevel analyses show that higher fertility has a significantly negative effect on longevity. For both women and men, the negative effects are stronger among the older cohorts and have reduced over time. Moreover, we find similar trends in the dynamic associations between fertility and longevity across four geographical regions in Europe. Findings and limitations of this study call for further investigations into the historical dynamics of multiple mechanisms behind the human evolution of ageing.

## 1. Introduction

Evolution theories since Darwin have posited that natural selection should lead to survival of the fittest. Such a selection on physical fitness is programmed to optimise the best performance of species survival in successive generations by balancing individual survival and reproduction [1]. Following Darwin’s legacy, many scholars in life history theory have sought to explain mechanisms behind the ageing process in human and other species [2]. An underpinning theory is the disposable soma theory, which pinpoints the “trade-off” between fertility and longevity. It argues that biological reproduction involves significant nutrition investments that could have been invested in somatic maintenance for survival [3]. Therefore, higher fertility comes with the costs of lower lifespan or higher late-life mortality.

To test the disposable soma theory in human species, Kirkwood and Westendorp used genealogical data from the British aristocracy and found significant trade-offs between fertility and longevity for women [4]. This seminal publication immediately triggered debates over the validity of their research design and whether the finding could be replicated in other human populations. Over more than two decades, empirical results on the topic remain inconclusive. Some studies found negative associations between human fertility and longevity (or mortality), while others claimed no significant or even positive association (see [5–7] for literature review). Meanwhile, most studies found that the association between fertility and longevity is asymmetric by parental sex, with only mothers bearing the survival costs of reproduction [4, 8–11]. In a nutshell, empirical results seem to differ across studies due to variations in time periods, populations in different regions, sample selection criteria, modelling methods, and the choice of relevant controls [6, 7, 12, 13].

In light of mixed empirical evidence on the relationship between fertility and longevity, emerging research has emphasised the role of environmental changes and variations in determining the magnitude of the trade-offs [14–16]. Our study extends this literature by examining how effects of human fertility on longevity vary over time in an historical period of rapid modernisation and epidemiological transition in Europe (from the seventeenth to the nineteenth centuries). We also highlight that such an historical trend could differ across populations in different geographical regions. We use crowdsourced genealogies from the FamiLinx data project [17] to derive our analytical sample, which comprises more than 185,000 women and men born between 1601 and 1910 in 16 European countries. We use a multilevel modelling strategy with censored Tobit regressions to address the unique issues of cohort-nested structures and sample selection in the study of pre-industrial human populations [12]. Our findings contribute to the empirical literature by showing the sex, time, and geographical heterogeneity in the fertility-longevity trade-offs using big data.

## 2. Theories and previous findings

In life history theory, a “trade-off” between fertility and longevity involves the allocation of metabolic resources and nutrients collected from external environment [18]. Given the necessity of balancing the limited resources invested in somatic maintenance for survival against reproductive functions, variations in the external environment are very likely to moderate physiological mechanisms that determine the trade-offs between fertility and longevity [19]. In line with this argument, an ecological hypothesis argues that the linkage between reproduction and survival partly depends on the early-life environmental risks to which individuals or species are exposed [16, 20, 21].

There is ample evidence showing that survival and reproduction are, to some extent, co-determined by environmental factors [22]. Experiencing environmental perturbations during the periods of development, such as drought or inferior nutritional conditions, has been found to impose a long-term effect on animals’ survival and reproductive fitness [23–26]. In non-human-species literature, researchers have shown that living in a more restricted survival condition, such as lower quality and higher density of the natal environment, is associated with higher fertility-longevity trade-offs [20, 27, 28]. Even for the most-studied cases of *Drosophila melanogaster* (common fruitfly) and *Caenorhabditis elegans* (roundworm), there is growing evidence that the trade-offs between fertility and longevity can be “uncoupled” by providing an artificially benign laboratory environment, which allows them to “realize their physiologically maximal possible investments into both survival and reproduction” [29].

In human studies, a more favorable early-life environment is found to be associated with higher reproductive success and a lower rate of late-life mortality [22, 30–33]. While these findings indicate the potential role of survival conditions in moderating the fertility-longevity relationship, only a few studies have investigated this hypothesis empirically in humans.

Different from studies of non-human species, where treatment can be randomly assigned to different groups through experiments, human studies rely primarily on observational data and use external shocks or systematic differences to gauge the variations in reproductive or survival conditions [15, 16, 21, 34, 35]. In addition, researchers often use data from historical populations to approximate a natural fertility scenario, which avoids estimating a spurious effect due to the manipulation of modern birth controls [36]. Thus far, three approaches have been used to test the moderating role of survival environment in the fertility-longevity relationship.

The first approach uses micro-level socioeconomic status as a proxy for material conditions [35, 37]. It assumes that a household’s higher socioeconomic status links directly to a preferable living condition. Using data from the historical Krummhörn population in Germany (1720–1870), Lycett et al. [35] observed the negative effect of fertility on longevity only for women in the lowest socioeconomic class (the landless household), while positive effects were found for women of higher socioeconomic status. Another study using parish records from pre-industrial Swedish populations also reported similar findings [37]: only landless women experienced higher mortality from having more children; neither men nor female freeholders/Crown tenants suffer from higher mortality due to having more children. Both studies have claimed that the extent to which fertility affects longevity is determined by the level of economic deprivation, and that women with the poorest living conditions may bear the highest survival costs of reproduction.

The second approach examines whether the fertility-longevity relationship depends on variations in environmental indices [15]. It assumes that a more favorable early-life survival condition should buffer the negative effect of fertility on longevity. Using demographic data from Lutheran churches in Finland (1751–1850), Nenko and colleagues [15] matched yearly information about local crop yields, spring temperatures, and infant mortality to their investigated sample based on individual birth cohorts. While their results did not show any evidence that women with a more unfavorable early-life environment suffered from higher mortality, they did find fluctuations in the fertility-longevity relationship by different birth cohorts, and they expressed their suspicion that women may have adjusted their numbers of pregnancies to cope with restricted environmental conditions.

Studies of these two approaches provide valuable insights by using fine-tuned proxies for survival conditions. However, data requirements for socioeconomic background or local-level indices inevitably restrict such investigations to a smaller population in a confined geographical region, such as the parish churches in Scania. Moreover, small sample size often plagues these studies’ statistical inference and leads to over-conservative conclusions, especially when investigating the fertility-longevity relationship among high-parous individuals (those with more than five children).

The third approach investigates changes in survival environments using a more inclusive proxy—birth cohorts [4, 14, 38]. This approach is justified from an evolutionary perspective if a population has experienced a systematic improvement or deterioration in survival conditions in a specific period during which cross-generational differences in ecological patterns could be observed [16]. In the European context, an ideal investigation period is from the seventeenth to the early twentieth centuries, when agriculture, industry, medicine, public health, and other sectors were making revolutionary-scale progress [39]. Using genealogical data from the British aristocracy, Westendorp and Kirkwood [4] found that the costs of reproduction on maternal longevity were higher for women born between 1500 and 1700 than for women born between 1701 and 1875. Kaptijn et al.’s [14] study of the population of the Netherlands population (cohorts 1850 to 1910) also found that the gaps in survival time between high parous women (more than five children) and women who bore no children have decreased over time. Focusing on a more modern population in Framingham, Massachusetts (in the United States), Wang et al. [38] found positive correlations between the number of children women born between 1893 and 1907 bore and their lifespan, and negative correlations for women born between 1908 and 1913. They argued that the more negative correlations in the younger cohorts (1908–1913) could be explained by their common experiences of environmental perturbations during their reproductive years, such as the Great Depression and the Second World War.

While using this cohort-proxy approach to test the environmental moderation hypothesis is straightforward and seems more practical than the first two approaches, there are several empirical challenges. One major criticism of the Westendorp and Kirkwood study [4] is that its findings of significant trade-offs before 1700 were based on a very small sample size, within which model estimates could have been overly sensitive to a handful of women with more than 15 children [40]. Conversely, Wang et al. [38] had a short observational window into birth cohorts (i.e., from 1893 to 1913), which largely decreases the validity of using cohort differences as proxies for environmental changes (because there simply are not enough changes). In this respect, Kaptijn et al. [14] provide more robust evidence by using a relatively large sample of 6,359 women spanning more than 60 cohorts in the Netherlands after 1850. However, whether we can infer the findings to other European populations or trace the evolution dynamics back to the early modern period (i.e., roughly before 1800) are still unknown.

In this study, we follow the third approach and hypothesise that the negative effect of fertility on longevity becomes less pronounced over time. To address the shortcomings of previous research, we use a large-scale dataset consisting of 16 European populations (calculated at the country level) to analyse the effect of human fertility on longevity over three centuries. Using a unified framework to study different populations, our empirical results provide scholars with a comparative perspective with which to validate life history theories and to develop meaningful debates on human evolution [5, 13, 36].

## 3. Material and methods

### 3.1. Data and sample

The data used in this study are derived from the FamiLinx data project (http://familinx.org/), which comprises millions of demographic records on individual life histories and family trees based on crowdsourced genealogies [17]. The original curators of the dataset collected the information following the terms and services of the genealogy-driven social network Geni.com and in cooperation with its parent company MyHeritage. We used the publicly available and fully anonymized version of the dataset, which does not contain personal user information, in compliance with the guidelines published by the FamiLinx project. The raw data covers a remarkable sample size of 86 million individual profiles and millions of network relationship files across the globe dating back to the fifteenth century [17]. We imported the raw files downloaded from the project website into a relational database using Python. To construct the analytical data, we followed Kaplanis et al.’s [17] method protocol to validate the values of individual records and to remove problematic pedigree patterns such as duplication, relational cycles, or multi-parents. S1 File provides the documentation for our steps in constructing the analytical samples.

An initial validation based on the completeness and reliability of responses restricts the raw sample size to around 3.5 million individuals across global populations (Sample 2 in S1 File). Individuals with incomplete information on sex, years and location of birth and death, and year of childbirth are excluded. We followed the common practice to exclude individuals born before 1600 to avoid issues of low data quality in genealogies preceding that date [4, 8, 17, 41]. While the selected genealogies come from more than 100 countries, a dominant proportion relates to individuals from North American (30%) and European (55%) countries [17]. To secure sufficient sample sizes for our multilevel models across cohorts and regions, we further restricted the analytical sample to the most populous 16 European countries in our dataset across four geographical regions (i.e., Scandinavia, Western Europe, Central Europe, and Southern Europe) (Sample 2.2 in S1 File).

For our research purposes, we focused on parous individuals, which restricts the analytical sample to women who gave birth to at least one child and the men who were assumedly the fathers of these children. This restriction rules out concerns regarding unreliable reports of nulliparous people in historical genealogies: due to the inheritance-centric nature of genealogies, under-reporting of children is common in low-parous individuals, especially for females in the side branches of genealogical trees [8, 42, 43]. Finally, we excluded extreme-age cases, that is, people who lived to be older than 100. Previous studies have shown that the prevalence of extremely long-lived people in genealogical data is prone to be unreliable and is inflated especially among incomplete genealogies [42]. After imposing this upper-end age restriction, the last cohort in our analyses comprises those born in 1910 and who died before 2010. In summary, the analytical sample includes 81,927 women and 103,642 men born between 1601 and 1910 and who had at least one child. S1 Table shows the numbers of cases across countries and birth cohorts.

Unlike in many studies, we did not exclude people who died before the end of their reproductive age periods (roughly before the age of 50). An arbitrary omission of pre-menopausal deaths results in under-estimation of the effect of fertility on longevity [8, 12, 13]. Firstly, the estimated trade-offs from the restricted sample are significantly biased toward zero given a pronounced selection on health and frailty [8, 12], because we may have excluded some physically robust individuals who could have survived to later ages were it not for the high costs of reproduction [13]. Meanwhile, frail women in the post-reproductive ages tend to have unrepresentative fertility patterns because only those whose reproductive costs are less than their frailty level could survive [13]. Second, there is also a selection on socioeconomic factors in the fertility-longevity relationship. In pre-industrial human societies in particular, the ability to survive to advanced age and afford a large family is largely determined by one’s wealth and social status [16, 36]. In this case, excluding cases of pre-menopausal deaths, especially before the nineteenth century, may have restricted our analysis to a subgroup in which the fertility-longevity trade-offs are buffered by benign economic conditions. To account for the systematic estimation bias resulted from these non-randomized sample selections, we followed Helle’s suggestion [12] and used the censored-normal Tobit regression with an age-truncated sample of European women and men to model the fertility-longevity trade-offs. Individuals who died before age 50 were left-censored and treated as having died exactly at age 50. More methodological justifications in favor of this method are discussed in the following sections.

### 3.2. Statistical models

In an analysis spanning over centuries, multiple factors related to historical changes in survival conditions may confound the relationship between human fertility and longevity. Among these factors, birth cohorts and their implicated “generation effects” are some of the most important factors that account for many unobserved heterogeneities, especially in exploring historical trends of vital statistics [44]. Individuals from the same birth cohort share unobserved attributes due to common influences of early-life survival conditions. In this regard, we argue that the historical trend in the relationship between fertility and longevity should be investigated from a hierarchical (multilevel) perspective, where observations of individuals are clustered (nested) in upper-level cohort groups. With this hierarchical structure, regression analysis based on a pooled-sample modelling method typically violates the assumption that random error terms should be uncorrelated across observations, leading to anti-conservative statistical inference. In addition, the pooled-sample method implies a uniform association between fertility and longevity across all observations, which violates our expectation that the effect of fertility on longevity could change over time. To address these issues, we need a multilevel modelling strategy. Specifically, the outcome variable *Y*_*ij*_ (i.e., longevity, measured as age at death) is modeled as a function of the lower-level predictor *x*_*ij*_ (i.e., fertility, measured as the number of all children born):

$$Y_{ij}=\beta_{j}^{I}+\beta_{j}^{x}x_{ij}+\epsilon_{ij}$$ (1)

where *i* indexes lower-level observations and *j* indexes upper-level clusters (i.e., cohorts). Here, we allow the regression intercept $\beta_{j}^{I}$ and the slope of the lower-level predictor *x*_*ij*_ to vary across clusters. These components are further decomposed in two cluster-level equations:

$$\beta_{j}^{I}=\;\gamma^{I}+\upsilon_{j}^{I}$$ (2)

and

$$\beta_{j}^{x}=\;\gamma^{x}+\upsilon_{j}^{x}$$ (3)

where *γ*^*I*^ and *γ*^*x*^ denote the average intercept and the average slope across cohort clusters. The remaining components are cluster-specific parameters, where the $\upsilon_{j}^{I}$ denotes cohort-specific variations from the average intercept and the $\upsilon_{j}^{x}$ denotes cohort-specific variations from the average slope. Substituting Eqs (2) and (3) into Eq (1) yields:

$$Y_{ij}=\underset{\begin{array}{c} \text{average}\\ \text{intercept} \end{array}}{\underset{︸}{\gamma^{I}}}+\underset{\begin{array}{c} \text{average}\\ \text{slope} \end{array}}{\underset{︸}{\gamma^{x}}}x_{ij}+\underset{\begin{array}{c} \text{cohort-specific}\\ \text{variations}\;\text{from}\\ \text{the}\;\text{avg}.\text{intercept} \end{array}}{\underset{︸}{\upsilon_{j}^{I}}}+\underset{\begin{array}{c} \text{cohort-specific}\\ \text{variations}\;\text{form}\\ \text{the}\;\text{avg}.\text{slope} \end{array}}{\underset{︸}{\upsilon_{j}^{x}}}x_{ij}+\underset{\begin{array}{c} \text{idiosyncratic}\\ \text{error}\;\text{term} \end{array}}{\underset{︸}{\varepsilon_{ij}}}$$ (4)

Based on Eq (4), we can use a fixed-effects modelling strategy with cluster-specific intercepts and slopes (FECS), where the $\upsilon_{j}^{I}$ and $\upsilon_{j}^{x}$ are estimated and controlled for. This method rules out all unobserved confounding factors attached to birth-cohort attributes while at the same time allows us to investigate the cross-cohort heterogeneity. The FECS is specified by adding *N-1* cohort dummies and their interactions with $\upsilon_{j}^{I}$ and $\upsilon_{j}^{x}$ into Eq (4):

$$Y_{ij}=\underset{\begin{array}{c} \text{average}\\ \text{intercept} \end{array}}{\underset{︸}{\gamma^{I}}}+\underset{\begin{array}{c} \text{average}\\ \text{slope} \end{array}}{\underset{︸}{\gamma^{x}}}x_{ij}+\sum_{j=1}^{N-1}\underset{\begin{array}{c} \text{cohort}\\ \text{fixed}\\ \text{effects} \end{array}}{\underset{︸}{\alpha_{j}\upsilon_{j}^{I}}}+\sum_{j=1}^{N-1}\underset{\begin{array}{c} \text{cohort-}\\ \text{specific}\\ \text{slopes} \end{array}}{\underset{︸}{\delta_{j}\upsilon_{j}^{x}}}x_{ij}+\underset{\begin{array}{c} \text{idiosyncratic}\\ \text{error}\;\text{term} \end{array}}{\underset{︸}{\varepsilon_{ij}}}$$ (5)

Where the $\alpha_{j}\upsilon_{j}^{I}$ is a vector of cohort-specific characteristics and the $\delta_{j}\upsilon_{j}^{x}$ denotes a vector of cohort-specific slopes. In summary, we use the FECS modelling method to estimate simultaneously the average intercepts and slopes (i.e., effects of fertility on longevity) and their cohort-specific variations. Because our dependent variable is left-censored at age 50, we use a Tobit linear regression instead of an ordinary least square (OLS) linear regression to estimate our parameters [45]. Tobit regressions use algorithms to recover the left-censored distribution of the limited dependent variable and utilize the full information from every individual in the sample. Monte Carlo simulation by Helle [12] has shown that the Tobit regression approach not only produces consistent regression estimates across replications with different sample sizes but also preserves high statistical power for research on the fertility-longevity trade-offs.

We cluster the sample into 13 cohort groups: 12 groups with an interval of 25 years from the seventeenth to nineteenth centuries (1601–1625, 1626–1650 …, 1876–1900) and one cohort group for those born between 1901 and 1910. All models control for individual age at last birth as a proxy for individual health status. Late childbirth is a signal of physical robustness, which also links to delayed ageing processes [13, 36, 46]. We control for individuals’ place of birth in four geographical regions: Western Europe, Scandinavia, Central Europe, and Southern Europe. In the analysis of regional comparison, we yield four regional subsamples and use the same multilevel Tobit regressions to model the fertility-longevity relationship. For statistical inference, we present robust standard errors adjusted for cohort heteroskedasticity using the Huber-White method.

Following our focal models are two sets of sensitivity analyses. The first ones use the same multilevel Tobit model with a subsample of individuals with more than two children, further conditioning on the mean inter-birth intervals (MBI) to account for some unobserved confounding effects related to unobserved socioeconomic factors. The second ones apply a similar FECS multilevel modelling strategy, but with a subsample of individuals who died after age 50 using an OLS linear regression, to estimate the effect of fertility on “post-reproductive” lifespan. In the following Section 3.3, we discuss the pros and cons of each model specification.

### 3.3. Model specification: A cautionary tale using DAGs

For researchers aiming at testing the theory-driven causal hypotheses using observational data, a cautionary selection of samples and independent variables is necessary. Following the methodological framework popular in modern epidemiology [47–50], we use the Directed Acyclic Graphs (DAGs) to illustrate our model specifications for the focal and the sensitivity analyses.

**Fig 1:**
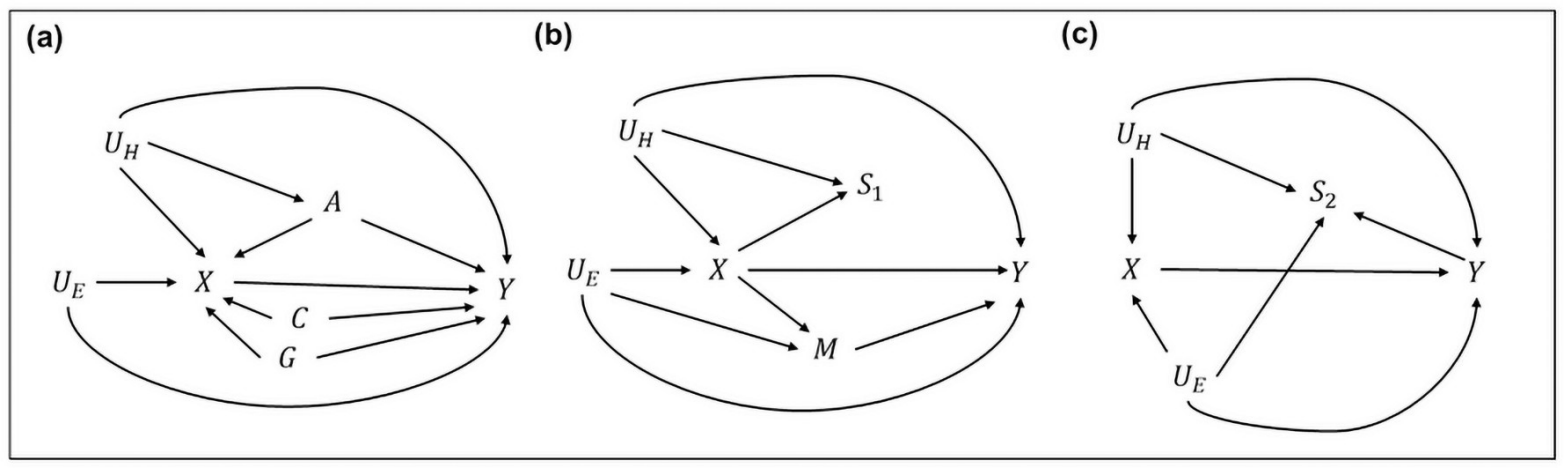
Directed Acyclic Graphs (DAGs) for the focal and the sensitivity analysis models. For clearer illustration, the DAGs in (b) and (c) do not include the paths related to A, C, G as presented in (a). These paths and variables, however, are applied across all three models.

Our focal model is illustrated in Fig 1a, which aims to estimate the effect of fertility *X* on longevity *Y* using the full sample without artificial sample selection on either *X* or *Y*. We condition on individual’s place of birth and birth cohorts to rule out their confounding effects that could cause spurious associations between *X* and *Y* (*X*←*C*→*Y* and *X*←*G*→*Y*). Aside from the cohort and geographical factors, two major factors confounding the causal relationship between *X* and *Y* are individual health status *U*_*H*_ and socioeconomic background *U*_*E*_ [5, 8]. Unfortunately, our data do not include variables that directly measure these two factors. As an alternative, we rely on proxy variables to partially account for the indirect confounding effects related to *U*_*H*_ and *U*_*E*_. We include age at last birth *A* as a proxy for the unobserved health status *U*_*H*_. Late childbirth is a signal of physical robustness, which directly links to higher reproductive fitness and delayed ageing process [13, 36, 46]. Conditioning on *A* partially adjust for the omitted variable bias by blocking out the two indirect confounding paths related to *U*_*H*_ in the backdoor (*X*←*A*←*U*_*H*_→*Y* and *X*←*A*←*U*_*H*_→*Y*) and the confounding path directly related to *A* (*X*←*A*→*Y*). However, the direct confounding path of *U*_*H*_ in the backdoor remains open (*X*←(*U*_*H*_*|A)*→*Y*), to the degree that the proxy *A* is not perfectly correlated with *U*_*H*_ [47, 48]. Therefore, our estimates on the effect of *X* on *Y* could still be biased given a strong confounding effect related to the unobserved *U*_*H*_ and *U*_*E*_.

One approach to account for more omitted variable biases is to include more proxies for *U*_*H*_ and *U*_*E*_. Previous studies have shown that poor socioeconomic status is associated with a slower reproductive rate for women, operationalized as having a longer mean inter-birth interval (MBI) [34, 51]. A relatively poor nutrition status due to the lack of resources in poor-status households could damage women’s physical fitness for intense reproduction, prolonging the first birth interval as well as the MBI [51, 52]. Moreover, some rich or noble women in preindustrial societies hired wet nurses to breastfeed their babies, resulting in a shorter MBI [53]. However, a very short MBI (i.e. intervals between pregnancies <6 months) could also lead to physical depletion, leading to a higher risk of maternal death [54]. Based on the discussion, we propose the first sensitivity analysis model by including the MBI variable *M* (and its squared term) as a proxy for the unobserved *U*_*E*_. However, by including *M*, this model is inevitably restricted to a subsample with at least two births, introducing a sample selection factor *S*_*1*_ based on the level of *X*. The DAG of this model is illustrated in Fig 1b. Conditioning on *M* partially adjusts for the omitted variable bias by blocking out an indirect confounding path related to *U*_*E*_ in the backdoor (*X*←*U*_*E*_→*M*→*Y*) via *M*. The direct confounding path of *U*_*E*_ in the backdoor remains open (*X*←(*U*_*E*_*|M)*→*Y*) to the degree that the proxy *M* is not perfectly correlated with *U*_*E*_. However, including *M* in a subsample analysis introduces several biases when estimating the effect of *X* on *Y*. Firstly, M is a collider in a backdoor path related to *U*_*E*_ (*X*→*M*←*U*_*E*_→*Y*). Conditioning on *M* will reopen this backdoor path, introducing an endogenous selection bias. Secondly, *M* is a mediator between *X* and *Y* (*X*→*M*→*Y*). Conditioning on *M* thus blocks this indirect causal path, leading to an overadjustment bias [49]. Thirdly, the required sample selection *S*_*1*_ for this analysis might lead to a sample selection bias [50]. Determined directly by people’s level of fertility *X*, being a sample in *S*_*1*_ also indicates a better reproductive fitness caused by a better health status *U*_*H*_. Selecting a subsample with more than 2 children is analogous to conditioning on *S*_*1*_ in the model, which could unblock a backdoor path from *X* to *Y* (*X*→*S*_*1*_←*U*_*H*_→*Y*) and further bias the estimation results.

Another popular approach aiming to reduce the omitted variable bias related to *U*_*H*_ is to select the analytical sample *S*_*2*_ by including only people who survived longer than the hypothetical reproductive age (roughly by age 50) [6, 9, 14, 35]. A common justification for the approach has been that the regression estimates of longevity on fertility could otherwise be “confounded” by an unobserved health deterioration that co-determines reproductive and survival lives [6, 35]. An underlying assumption is that including women who died before menopause would mask the fertility-longevity trade-off that should have been observed because pre-menopausal death may lead to incomplete reproductive lifespan and thus depress fertility. However, this assumption does not always hold in empirical studies. For example, Westendorp and Kirkwood [4] show that the longest-lived groups in pre-industrialized England had the lowest fertility comparing to others; women who died after age 90 had lower fertility than women who died around age 40. In our FamiLinx sample, the fertility differences between early-death women and later-death women have also been small, particularly before the nineteenth century (see S1 Fig). Since the empirical foundation supporting the exclusion of early-death cases is weak, evolutionary scholars should instead consider other modelling strategies that can utilize the full information from both the pre- and post-menopausal sample to avoid unnecessary sample selection [12, 15].

More crucially from the theoretical perspective, the costs of reproduction on survival manifest not only after the end of one’s reproductive period but also before it [55]. Indeed, among other long-lived mammal species, early reproductive costs on either later reproduction or survival manifest even more during their reproductive period [56, 57]. Imposing the age selection criteria *S*_*2*_ without correcting for the mortality selection issue could bias the estimation results in many studies [6, 11, 35, 37], even if their inferences to cover only the “post-reproductive” or “late-life” longevity and mortality have already been restricted (see [8, 12, 13] for discussions). Fig 1c illustrates the DAG of this model. Whether an individual is studied in *S*_*2*_ is determined directly by their age at death *Y*. Being selected to *S*_*2*_ manifests a better survival fitness due to better health conditions (*U*_*H*_→*S*_*2*_). Moreover, survival beyond age 50 in pre-industrial populations requires affluent material resources in early life, reflecting the living condition of rich socioeconomic groups (*U*_*E*_→*S*_*2*_). Given such relationships, selecting a subsample of post-reproductive people is analogous to conditioning on *S*_*2*_ in the model, which could unblock two backdoor paths from *X* to *Y* (*X*←*U*_*H*_→*S*_*2*_←*Y* and *X*←*U*_*E*_→*S*_*2*_←*Y*) and further bias the estimation.

In summary, we argue that Model (a) in Fig 1 is the benchmark approach among the three to test whether the hypothesized negative effect of fertility on longevity exists and changes over time, due to its relatively small sample selection and collider biases comparing to the other two. Meanwhile, we provide estimation results from Models (b) and (c) as sensitivity analyses, which are informative by comparison in terms of how health- and wealth-related sample selection, overadjustment, and omitted variable biases could change and even alter the direction of the trade-offs observed in our focal model. Firstly, Model (b) might perform better during a historical period of high natural fertility (i.e., reduce the sample selection bias) and low risk of birth-related death (i.e., reduce the overadjustment bias). In Europe, this roughly refers to an early-industrial period from the mid- to the late-nineteenth century before introducing the population-level contraception [58]. Secondly, Model (c) might better during a historical period of longer lifespan when survival beyond 50 years old is common regardless of health and socioeconomic status, which also refers to the period after the mid-nineteenth century. Finally, we can use the estimation results from Model (c) to check for the risk of reverse causality in Model (a). That is, it might be one’s lifespan that determines her/his fertility rather than vise versa. Under the assumption that a longer lifespan positively associates with higher fertility, we might expect an even stronger negative effect of fertility on longevity in Model (c) comparing to Model (a) if the reverse causality is dominating. This is because Model (c) has largely ruled out the risk of reverse causality by focusing only on the post-reproductive subsample, thereby exempting from the positive association between fertility and lifespan. On the other hand, if a stronger negative effect in Model (a) comparing to Model (c) is observed, we might expect that the negative causal effect of fertility on longevity is dominating even among those with earlier death.

## 4. Results

### 4.1. Descriptive statistics

Table 1 presents descriptive statistics of variables of this study; the numbers of cases in each country and region across birth cohorts are presented in S1 Table. For simplicity of presenting the historical trend, we directly interpret the dynamic results from a cohort perspective. Focusing on women, Table 1 shows that women’s mean age at death has increased over time, from 61.2 in the early 1600s to 77.6 in the early 1900s. The maternal mean number of children grew from 2.6 children in the early 1600s to around 4.4 children in the mid-1800s and then declined again to around 3.8 in the early 1900s. Meanwhile, maternal age at last birth also increased from 31.3 years to around 35 years between 1601 and 1850 and then declined to less than 30 years in the twentieth century. This number is comparable to the age range of last birth from 31 to 40 years in historical human populations [59]. Women’s mean inter-birth interval (MBI) remained around 3.5 years for most of the historical cohorts until its decline in the late-19^th^ century during the demographic transition. The number is comparable to Nenko and colleagues’ finding of 43.13 months (i.e., 3.59 years) in pre-industrial Finnish populations [34].

**Table 1:** Descriptive statistics of life-history traits of samples by sex.

	Obs.	Age at death		N of children		Age at last birth		MBI (for people with more than 2 children)	
		Mean	SD	Mean	SD	Mean	SD	Mean	SD
***Women***	*81*,*927*	*66*.*45*	*17*.*12*	*3*.*75*	*3*.*22*	*33*.*29*	*7*.*21*	*3*.*22*	*2*.*36*
1601–1625	888	61.15	16.36	2.63	3.01	31.28	8.25	3.55	3.02
1626–1650	1,506	61.37	16.12	2.61	3.02	31.50	7.93	3.69	3.13
1651–1675	1,858	61.45	16.38	2.70	2.91	32.31	7.62	3.50	2.77
1676–1700	2,940	63.45	15.69	3.05	3.12	33.38	7.47	3.59	3.06
1701–1725	4,396	63.28	15.80	3.13	3.18	33.48	7.34	3.61	2.86
1726–1750	5,915	64.19	15.78	3.18	3.16	33.49	7.24	3.60	2.71
1751–1775	7,807	63.76	15.87	3.29	3.12	33.74	7.14	3.65	2.65
1776–1800	9,410	63.63	16.66	3.63	3.21	33.79	7.26	3.60	2.44
1801–1825	11,115	64.64	17.27	3.96	3.31	34.45	7.21	3.48	2.39
1826–1850	12,135	66.53	17.54	4.37	3.46	34.74	7.04	3.31	2.20
1851–1875	11,270	68.59	17.18	4.30	3.29	33.51	6.95	3.11	2.00
1876–1900	9,490	73.24	16.62	3.88	2.84	30.84	6.36	2.62	1.97
1901–1910	3,197	77.59	15.32	3.79	2.49	28.89	5.98	1.78	1.66
***Men***	*103*,*642*	*66*.*73*	*14*.*88*	*3*.*83*	*3*.*39*	*37*.*83*	*8*.*91*	*3*.*27*	*2*.*64*
1601–1625	1,896	64.12	13.95	2.60	3.09	37.47	10.25	3.78	3.54
1626–1650	2,685	63.46	14.79	2.78	3.31	37.53	9.81	3.44	3.18
1651–1675	3,090	63.14	15.06	2.81	3.15	37.60	9.50	3.65	3.06
1676–1700	4,324	63.55	14.12	3.09	3.33	37.68	9.64	3.86	3.66
1701–1725	6,697	64.73	14.38	3.20	3.38	37.91	9.35	3.79	3.30
1726–1750	8,505	65.26	14.03	3.31	3.35	38.55	9.54	3.90	3.33
1751–1775	10,402	64.75	14.13	3.42	3.33	38.51	9.20	3.73	2.94
1776–1800	11,556	64.90	14.81	3.87	3.47	38.15	9.06	3.60	2.72
1801–1825	13,942	66.28	15.13	4.24	3.55	39.31	9.02	3.50	2.62
1826–1850	14,120	67.77	14.97	4.62	3.63	39.38	8.61	3.32	2.34
1851–1875	12,930	69.17	14.77	4.36	3.34	37.55	7.80	3.10	2.08
1876–1900	10,206	71.27	14.88	3.90	2.80	34.26	7.06	2.44	1.92
1901–1910	3,289	72.84	14.17	3.81	2.29	32.72	6.59	1.66	1.55

Descriptive results show that women in our analytical sample had a relatively long life expectancy compared to the population-level average during their historical periods [60]. This is because we only selected parous individuals into our analysis, thereby excluding a large proportion of premature deaths in historical populations. At first glance, the sampled women’s cohort fertility rates (measured by the average number of children) are relatively low. However, our finding of the low fertility in the seventeenth century and the long period of fertility increase before the nineteenth century is comparable to the fertility trend in historical England [61] (see S2 Fig). In summary, several demographic indices calculated from our FamiLinx genealogical sample represent well the population-level trend in human history, particularly among the pre-industrial populations [17].

### 4.2. Fertility has a negative effect on human longevity, especially for women

Table 2 presents the effect of childbirth on women’s longevity. Beginning with a pooled-sample Tobit regression without control variable for women (Model 1), an additional child is associated with a 0.392 year increase in maternal lifespan (*p* < 0.001), which implies a positive effect of fertility on women’s longevity. After controlling for women’s age at last birth (Model 2), the positive association between fertility and longevity has weakened but remained statistically significant. Model 3 further accounts for the multilevel structure by specifying the cohort fixed effects. The estimated effect turns negative: having an additional child significantly reduces maternal lifespan by 0.193 years (about 70 days, *p* < 0.001).

**Table 2:** Effect of an additional child on maternal lifespan (in years).

	Model 1			Model 2			Model 3		
	Pooled Tobit model w/o control			Pooled Tobit model			Multilevel Tobit model		
	Β	*std*. *err*.	*p*	β	*std*. *err*.	*p*	β	*std*. *err*.	*p*
N of children	0.392	0.017	<0.001	0.101	0.020	<0.001	-0.193	0.020	<0.001
Age at last birth				0.240	0.009	<0.001	0.403	0.009	<0.001
Place of birth	Yes			Yes			Yes		
Cohort fixed effects	No			No			Yes		
N (Total)	81,927			81,927			81,927		
N (Uncensored)	66,238			66,238			66,238		
N (Censored)	15,689			15,689			15,689		

Note: Models 1 & 2 use pooled-sample Tobit regressions, controlling for individuals’ place of birth in four regions. Model 3 uses the multilevel Tobit regression model conditioning on cohort fixed effects. All models use the full sample of 81,927 parous women.

We find similar patterns for men in Table 3. Without considering cohort heterogeneities, both Model 1 and Model 2 show that men’s fertility are positive associated with longevity (Model 1: β = 0.393, *p* < 0.001; Model 2:: β = 0.105, *p* < 0.001). After accounting for the cohort differences in Model 3, we found a significantly negative effect of fertility on paternal longevity (β = −0.078, *p* < 0.001).

**Table 3:** Effect of an additional child on paternal lifespan (in years).

	Model 1			Model 2			Model 3		
	Pooled Tobit model w/o control			Pooled Tobit model			Multilevel Tobit model		
	β	*std*. *err*.	*p*	β	*std*. *err*.	*p*	β	*std*. *err*.	*p*
N of children	0.393	0.012	<0.001	0.105	0.014	<0.001	-0.078	0.014	<0.001
Age at last birth				0.217	0.005	<0.001	0.300	0.006	<0.001
Place of birth	Yes			Yes			Yes		
Cohort fixed effects	No			No			No		
N (Total)	103,642			103,642			103,642		
N (Uncensored)	87,672			87,672			87,672		
N (Censored)	15,970			15,970			15,970		

Note: Models 1 & 2 use pooled-sample Tobit regressions, controlling for individuals’ place of birth in four regions. Model 3 uses the multilevel Tobit regression model conditioning on cohort fixed effects. All models use the full sample of 103,642 parous men.

**Fig 2:**
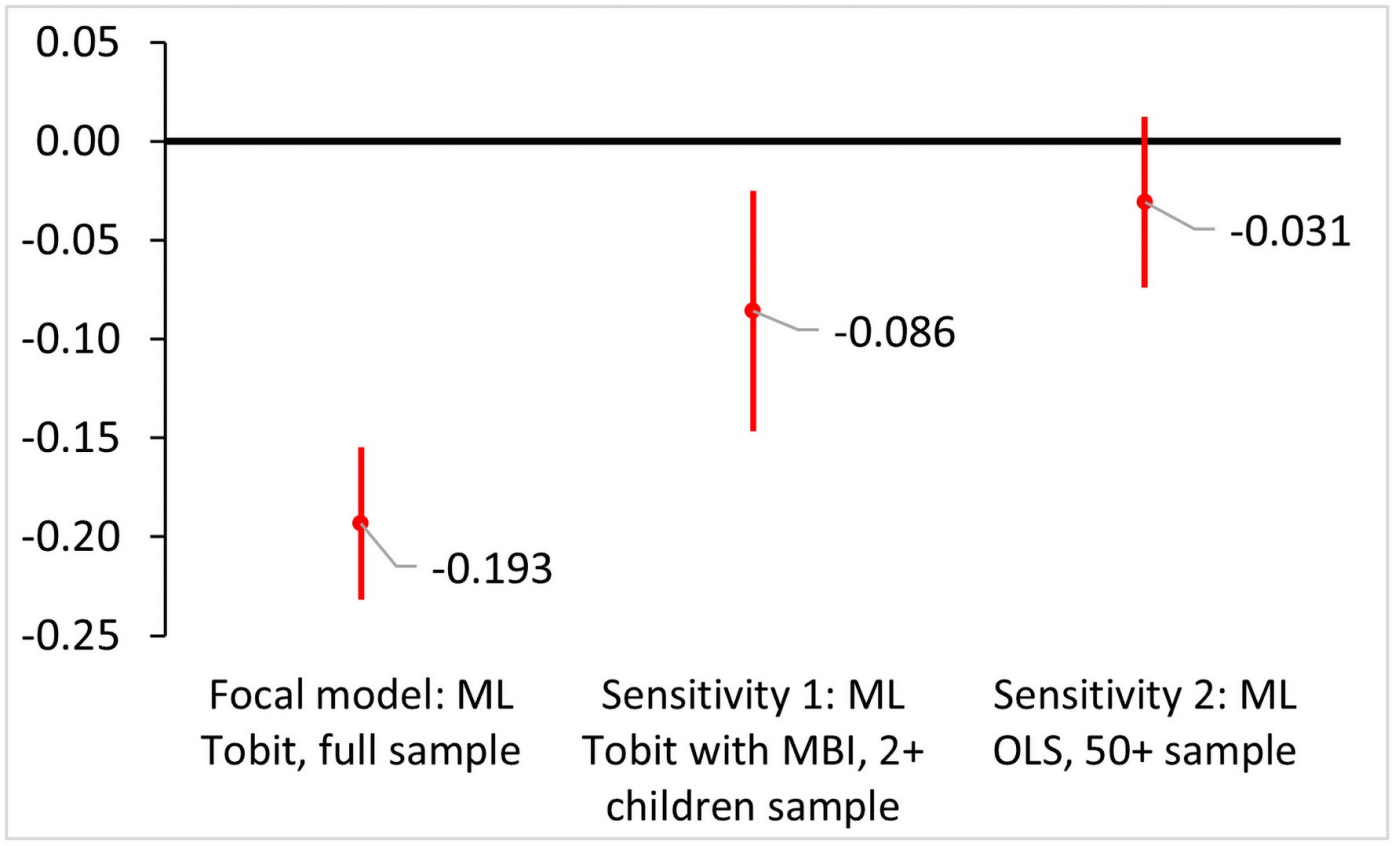
Effect of an additional child on maternal lifespan (in years) across different model specifications. Point estimates of the fertility effects with 95% confidence intervals are presented. The “Focal model” refers to the estimation results of Model 3, Table 2. The “Sensitivity 1” refers to the estimation results of Model S1, S2 Table. The “Sensitivity 2” refers to the estimation results of Model S2, S2 Table.

We perform sensitivity analyses using different model specifications discussed in Fig 1b and 1c. Sensitivity analyses for women’s and men’s fertility-longevity trade-offs are presented in S2 and S3 Tables as supporting materials. Focusing on women, we plot in Fig 2 the estimation results from our focal model and the first two sensitivity analyses. The first sensitivity analysis shows that by adding women’s MBI to the multilevel Tobit model and restricting the sample to higher-parous women, the negative effect of fertility on maternal longevity has reduced to –0.086, yet it remains statistically significant (*p* = 0.006). Results from the second sensitivity analysis using a multilevel OLS model with later-death women show an even smaller and statistically insignificant negative effect of fertility on maternal longevity (β = −0.031, *p* = 0.188). This result echos Doblhammer and Oeppen’s selection-corrected survival analyses [8] and Helle’s simulation [12] that the estimated fertility-longevity relationship from the age 50+ subsample tends to bias toward zero if systematic sample selection biases exist.

In summary, our results across the focal and the sensitivity analyses indicate that the theory-predicted trade-off between fertility and longevity is relatively robust for women. For men, the negative relationship found in Table 3 is less robust across the sensitivity analyses in S3 Table, with two of the sensitivity models estimate either a nearly zero effect or a positive effect of children on paternal lifespan.

### 4.3. Changing magnitudes of the trade-offs and regional heterogeneity

**Fig 3:**
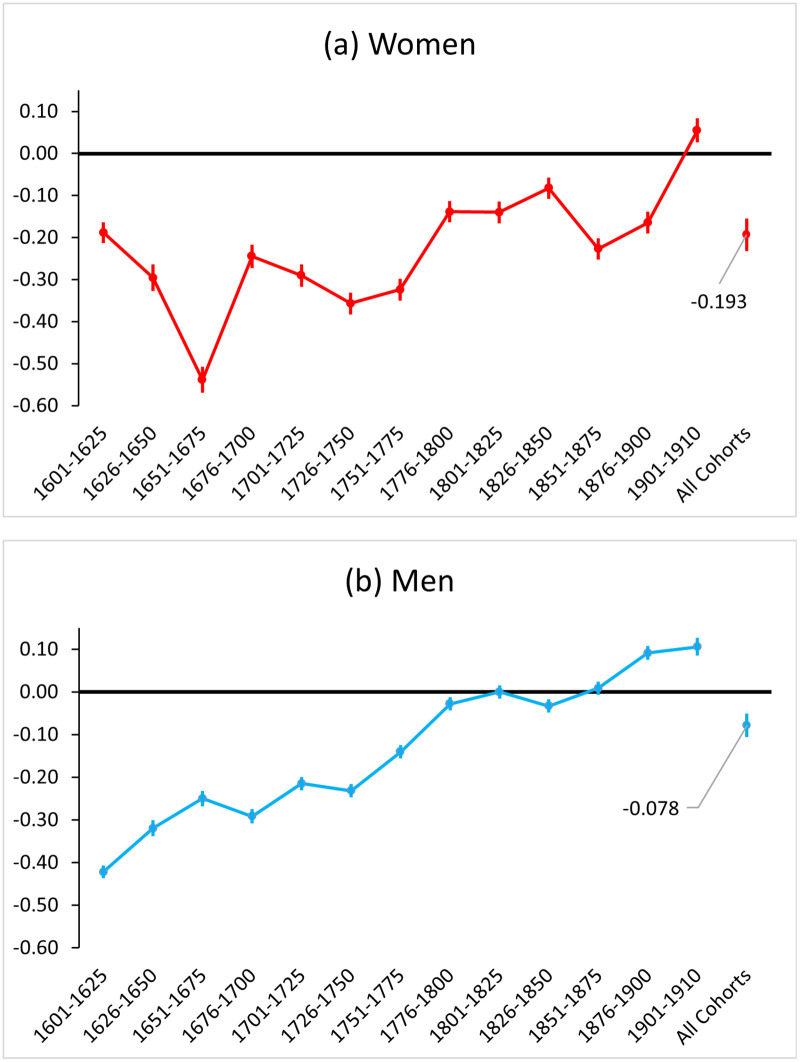
Effect of an additional child on parental lifespan (in years) by cohorts and sex. Point estimates of the fertility effects on lifespan with 95% confidence intervals are presented. Results are estimated using the female and male full samples and the FECS multilevel Tobit regression models, controlling for age at last birth and individuals’ place of birth.

Fig 3 presents the cohort-specific effects of childbirth on parental lifespan. In general, our prediction that the trade-offs between fertility and longevity should weaken over time is confirmed. A key historical period is observed around the late eighteenth century. For women born before 1800, fertility had much stronger negative effects on longevity compared to women of later cohorts. The negative effect turned positive for women born after 1900. We found a similar pattern of declining and reversing trade-offs for men; the effects of fertility on male longevity declined continuously before the nineteenth century and have been positive since 1875.

In the sensitivity analyses (results shown in S3 Fig), we found that the negative effect of fertility on longevity in historical times and their declining trends are flattened if we consider only people with more than 2 children or older than 50 in the multilevel models. Focusing on women, the estimated effects have been significantly more negative in the focal model comparing to the two sensitivity models before 1800. However, the estimated effects are converging over time and have reversed in the late-nineteenth century. According to our discussion in Section 3.3, this convergence indicates that health- and socioeconomic-related selections on both fertility and longevity were strong in pre-industrial human populations, especially before the nineteenth century. The negative effects of the 1901–1910 cohorts in both sensitivity analyses also contradict the focal model’s finding of a positive effect. For women of this specific cohort, the estimation results of the negative effects from both sensitivity models could be more reliable than that from the focal model because these women grew up during a historical period when having more than two children and survival to post-reproductive ages have been less selective based on their health and socioeconomic status. Also, for women born after 1900, the more negative effect of fertility on longevity in the second sensitivity model comparing to the focal model indicates that applying the Tobit regression method in a more modern period might bear the risk of introducing reverse causality.

In summary, results from the sensitivity analyses for women support our expectation in Section 3.3 regarding the direction of sample selection bias in the corresponding historical periods. Still, we want to highlight that the predicted weakening trend of the fertility-longevity trade-off over pre-industrial time remains largely robust across sensitivity analyses, except for the more modern period after 1850.

**Fig 4:**
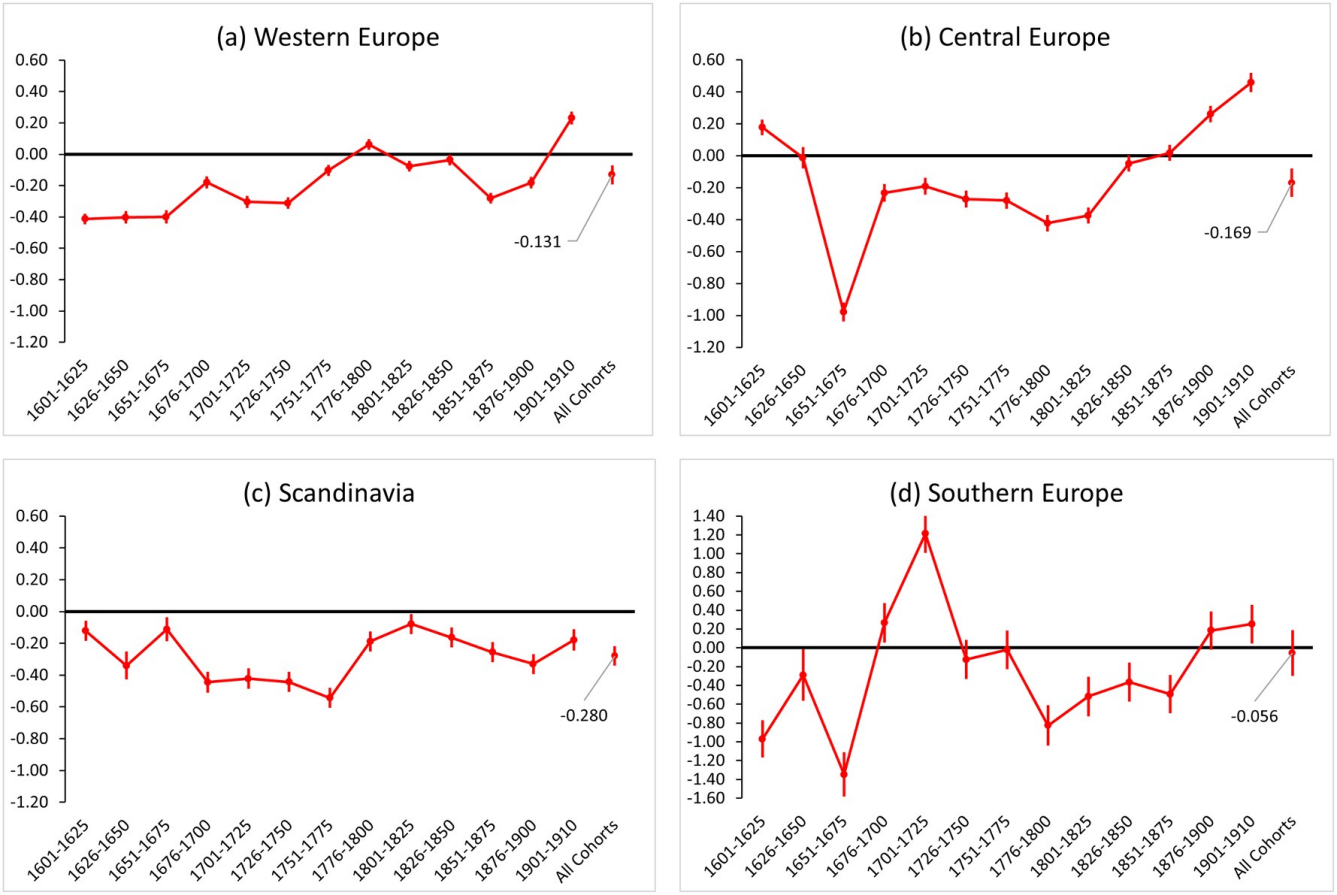
Effect of an additional child on maternal lifespan (in years) by cohorts and regions. Note: Point estimates of the fertility effects on lifespan with 95% confidence intervals are presented. Women’s results are estimated using four regional subsamples and the FECS multilevel Tobit regression models, controlling for age at last birth.

Finally, we explore whether the association between fertility and longevity and its historical trend differ by populations in different regions. Focusing on women, Fig 4 presents the results based on multilevel Tobit models. We find negative effects of children on lifespan for all female populations across geographical regions in Europe. The largest effect is in Scandinavia (β = −0.280, *p* <0.001), while the smallest effect is in Southern Europe (β = −0.056, *p* = 0.657). In Western and Central Europe, we find similar trends: the negative effect of fertility on longevity declines over cohorts. In Scandinavia, the effect in the early 1800s is much smaller than in the 1700s (although it increased again for later cohorts). The trend in fertility effects for women in Southern Europe is relatively volatile in the earlier cohorts (before 1750) due to the relatively small sample size. For Southern European women born after 1800, we also find a similar pattern of declining and even reversing trade-offs. In summary, except for the more volatile results reported in Southern Europe before 1750, our findings for female populations across regions underscore both the heterogeneity of the effect size and the homogeneity of the declining effect trend over time.

## 5. Conclusion and discussion

Using crowdsourced genealogy data of populations across Europe, we found that having more children may significantly reduce parental lifespan, especially for women. These findings generally support the prediction of disposable soma theory, which argues that multiple reproductions come with significant survival costs especially for females due to the depletion of physical resources that could have been utilised for somatic maintenance [4].

Further investigation into cohort-specific patterns adds nuance to the claimed “trade-offs” between fertility and longevity. Life history theories argue that the fertility effect on longevity depends largely on individuals’ survival environment: the “trade-offs” should be more prominent under constrained survival conditions [14, 15, 19, 35]. In European history, the improvement of material conditions since the seventeenth century has largely lifted the material constraints on what humans need for both parenting and survival [39]. These external changes may “shift the optimal balance that was [once] established through natural selection under previous conditions” [19]. Using the historical trend as a proxy, our multilevel models found that the negative effect of childbirth on women’s lifespan shrank remarkably over time, especially during the seventeenth and eighteenth centuries. Once again, a similar trend of declining fertility effect over time was also observed for men.

Findings of the synchronised trends in the fertility-longevity relationship for both sexes leave us with an unresolved puzzle: Is the negative association between fertility and longevity primarily a consequence of physiological trade-offs, as the disposable soma theory suggests [3, 4], or does it speak more to the existence of social factors that co-determine human survival and reproduction? If the relationship between human fertility and longevity is dominated by physiological trade-offs, we should expect significantly higher costs of reproduction for women than for men [8, 37, 62]. In general, our results confirmed such a sex gap when all cohorts and regions were pooled together for estimation. However, when examining the cohort-specific patterns in Fig 3, the negative fertility effects for men born before 1750 almost parallel the effects for women. How these effects are evolving over time is also similar for both sexes. Following previous research, we speculate that the similarity between men and women indicates the existence of social and economic costs of children, such as the income burden of childrearing [8, 63], which simultaneously determine people’s fertility and lifespan regardless of gender. In historical populations in particular, these socio-economic factors may play a crucial role in affecting humans’ health and survival [34, 35, 37].

Finally, our analyses show that the negative effect of fertility on longevity may differ across female populations. While there are heterogeneities in the effect size, what really surprises us is the similarity in how these effects are evolving over time. This across-population trend strengthens the theoretical claim that early-life survival conditions could moderate the magnitude of trade-offs between fertility and longevity [15, 16, 19]. Using a multilevel method and a harmonised dataset that covers multiple populations, this study contributes to quantifying regional and cohort differences/similarities in the fertility-longevity relationship. Our findings call for more studies in this comparative strand, as this methodological perspective is crucial for theory building in evolution science [5, 13].

We should make note of some limitations of this study. First, the extensive sample size of the FamiLinx dataset comes at the cost of missing important variables that are relevant when studying the causality between fertility and longevity. It is especially unfortunate that we cannot control for individual socioeconomic background, which could confound the estimation of trade-offs [13–15, 35]. Like other studies for historical populations, we also cannot control for individual heterogeneity in health. As an alternative, we follow previous research and use individual age at last birth as a proxy for physical robustness [13, 36, 37]. This proxy makes more sense in natural fertility environments where social selections on birth timing can be largely ignored [59]. We also consider women’s pace of reproduction, measured by MBI, as another proxy for socioeconomic status in the sensitivity analysis. However, including proxy variables is not the panacea for ruling out the omitted variable biases because the unobserved confounding factors remain. In some cases, including proxies even introduces new sources of bias, as we have discussed in Section 3.3. Given these issues related to unobserved variables, we should interpret the modelling results cautiously, especially for more recent cohorts where individual social and health conditions are more heterogeneous than in pre-industrial times.

Second, as presumably with all genealogy data, the FamiLinx dataset has a retrospective sample selection bias on both fertility and longevity [41, 43]. People with longer lifespans and more offspring have a higher probability of being recorded [43]. This sample characteristic could negatively bias the correlation between longevity and fertility for both men and women. A gender-based sample selection where men are overrepresented is another common characteristic for inheritance-centric genealogies [43]. This issue is also reflected in our FamiLinx sample, where the male sample comprises 26% more observations than the female sample. However, since we model the male and female trade-offs separately, this gender-based selection should not bias the estimation results. What is worth noticing is that the most complete and reliable female records in ancient genealogies tend to overrepresent rich or noble families [41, 43]. Given this data characteristic, one should be cautious in inferring our findings at the population level, particularly regarding the larger trade-offs for females before the eighteenth century.

Third, our research design is formulated to test the phenotypical trade-off between fertility and longevity and its historical dynamics predicted by the disposable soma theory. However, due to data limitations, we are not able to discuss the health or socioeconomic mechanisms lying behind. Recent studies have offered rich insights into disentangling various mechanisms behind the survival cost of reproduction, including both bio-physiological models and social explanations [62]. With advanced design using appropriate reference groups as control (e.g., siblings, twin birth, sex composition of children) or considering heritabilities and genetic correlations, future research using observational data could extend our multilevel models to investigate whether and to what extent the relative importance of different mechanisms has changed over time.

## Supporting information

S1 File Data curation and sample construction using the FamiLinx data. (DOCX)

S1 Table Sample cases by region, country, and birth cohorts. (DOCX)

S2 Table Sensitivity analyses of the effect of an additional child on maternal lifespan (in years). (DOCX)

S3 Table Sensitivity analyses of the effect of an additional child on paternal lifespan (in years). (DOCX)

S1 Fig Comparing cohort fertility between subsamples with pre-menopausal and post-menopausal death. (DOCX)

S2 Fig Crude Birth Rate (CBR) in England and Cohort Fertility Rate (CFR) in our FamiLinx sample. (DOCX)

S3 Fig Effect of an additional child on lifespan (in years) by birth cohorts, results from different models and samples. (DOCX)
