# Supplementary material for: Examining the trade-offs between human fertility and longevity over three centuries using crowdsourced genealogy data
Source: PLoS One. 2021 Aug 5;16(8):e0255528. doi: 10.1371/journal.pone.0255528 (PMC8341544; doi:10.1371/journal.pone.0255528)
Supplement: S1 File — (DOCX) [file pone.0255528.s001.docx]

# **S1 File. Data curation and sample construction using the FamiLinx data**

## Import data

We obtained the anonymized and openly accessible dataset from <https://familinx.org>. The dataset comprises two tab-separated files, one containing profile information for each observed individual (75 attributes per observation, see the official documentation for details) and the other containing data on parent-child relationships. After downloading the dataset from the website, we imported the data into a relational database using Python. During the import, we validated the imported records to ensure that they are correctly formatted.

## Sampling

After importing the dataset and based on its documentation (Kaplanis et al., 2018), we defined subsets of the data based on different quality criteria. The criteria set in the definitions pertain to attributes of interest to our analyses. Since many of the records comprising the full dataset, focusing on (partially) complete records is an important prerequisite to our analysis.

### Sample 1: Raw sample with complete records

The first sample is the baseline sample that contains complete records regarding the core attributes required to analyze the longevity of the subjects comprising the dataset. Records belonging to this sample have to fulfill the following requirements:

- `birth_year` is specified as a four digit number between 1600 and 1910

- `death_year` is specified as a four digit number between 0 and 2016

- `gender` is specified as either male or female

Note that we remove individuals born before 1600 or after 1910 to increase reliability and avoid ascertainment bias (cf. Kaplanis et al., 2018). This sample allows us to study the longevity of individuals based on their age of death, which we compute approximately by subtracting the year of their birth from the year of their death.

### Sample 2: Global sample with complete and reliable response

The second sample is based on the first sample and has stricter requirements that extend to additional attributes. Records belonging to this sample have to fulfill the following criteria:

- actor belongs to sample 1

- `birth_day` is specified as a number between 1 and 31

- `birth_month` is specified as a number between 1 and 12

- `birth_date_circa` is 0

- `birth_location_country` is not missing

- `birth_location_latitude` is not missing

- `birth_location_longitude` is not missing

- `death_day` is specified as a number between 1 and 31

- `death_month` is specified as a number between 1 and 12

- `death_date_circa` is 0

- `death_location_country` is not missing

- `death_location_latitude` is not missing

- `death_location_longitude` is not missing

This sample, while being more restrictive, allows us to calculate the correct age at death based on the full and precise birth and death dates. Further, by requiring records to have complete geographical assignments for their birth and death locations, we can consider geographical attributes in our analysis of this subset. In addition, it allows us to define geographically restricted subsets, which we discuss in the following. We use this sample (also referred to as the analytical sample) as the foundation for our analyses and the curation of regional samples.

### Sample 2.1: Full European sample with complete and reliable response

Sample 2.1 is a regional sample comprising all actors born in the EU. The list of EU countries we used is identical to the one used by Kaplanis et al. (2018):

- actor belongs to sample 2

- `birth_location_country` has one of the following values:

"Albania", "Austria", "Bosnia and Herzegovina", "Belgium", "Bulgaria",

"Switzerland", "Cyprus", "Czechia", "Germany", "Denmark", "Estonia",

"Spain", "Finland", "Faroe Islands", "France", "United Kingdom", "Great Britain",

"Gibraltar", "Greece", "Croatia", "Hungary", "Ireland", "Iceland", "Italy",

"Lithuania", "Luxembourg", "Latvia", "Monaco", "Republic of North Macedonia",

"Netherlands", "Norway", "Poland", "Portugal", "Romania", "Sweden", "Slovenia",

"Slovakia", "San Marino", "Holy See"

- OR `birth_location_country_code` has one of the following values:

"AL", "AT", "BA", "BE", "BG", "CH", "CY", "CZ", "DE", "DK", "EE", "ES", "FI", "FO", "FR", "GB", "GI", "GR",

"HR", "HU", "IE", "IS", "IT" ,"LT", "LU", "LV", "MC", "MK", "NL", "NO", "PL", "PT", "RO", "SE", "SI", "SK",

"SM", "VA"

### Sample 2.2: 16 European countries sample with complete and reliable response

Sample 2.2 is a further restricted regional sample used for our analysis, which comprises all actors born in 16 populous European countries during the historical period of investigation. The list of EU countries we used is identified as follows:

- actor belongs to sample 2.1

- `birth_location_country` has one of the following values:

"Austria", "Belgium", "Switzerland", "Czechia", "Germany", "Denmark",

"Spain", "Finland", "France", "United Kingdom", "Great Britain",

"Italy", "Netherlands", "Norway", "Poland", "Portugal", "Sweden",

- OR `birth_location_country_code` has one of the following values:

"AT", "BE", "CH", "CZ", "DE", "DK", "ES", "FI", "FR", "UK", "GB",

"IT", "NL", "NO", "PL", "PT", "SE"

## Filters

In addition to the rules implied by the samples, we applied the following restrictions to the Sample 2.2 before conducting our analyses:

- `age_at_death` must be >= 0

- `age_at_death` must be <= 100

- `age_of_birth_first_child` must be >= 10 and not NA

- `age_of_birth_first_child` must be <= 70 and not NA for male actors

- `age_of_birth_first_child` must be <= 55 and not NA for female actors

- `number_of_children` must be <= 20 and >= 1

These additional filters are applied to exclude cases with missing values in key life-history variables. Unreliable reproductive records including extremely early and late childbirth and extreme-age cases who lived more than 100 years are also excluded. Finally, for our purpose of investigating the reproductive cost of survival, we exclude nulliparous cases and cases with an extremely high number of children.

## Sample sizes by gender after each selection criteria

Table A1 presents the sample sizes in each step of the nested sample structure. The final sample used for our formal analyses is “Sample 2.2 with Filters”.

| Samples | N(Total) | % of the previous sample (Total) | N(Female) | % of the previous sample (Female) | N(Male) | % of the previous sample (Male) |
| --- | --- | --- | --- | --- | --- | --- |
| Sample 1 | 16,363,977 | 100.0% | 7,464,007 | 100.0% | 8,899,970 | 100.0% |
| Sample 2 | 3,489,040 | 21.3% | 1,562,450 | 20.9% | 1,926,590 | 21.6% |
| Sample 2.1 | 1,153,753 | 33.1% | 515,764 | 33.0% | 637,989 | 33.1% |
| Sample 2.2 | 1,036,076 | 89.8% | 464,779 | 90.1% | 571,297 | 89.5% |
| Sample 2.2 with Filters (Final sample) | 185,569 | 17.9% | 81,927 | 17.6% | 103,642 | 18.1% |

In general, the gender-based sample selection where men are overrepresented in inheritance-centric genealogies also applies to our FamiLinx data. However, by imposing the same filtering criterion, sample reductions (in percentage scales) in each step across the male and the female samples are similar. This result implies that the “exclusion” of actors from our analytical samples is not driven by gender-based selection. It is more likely that the exclusion is driven by systematic missing of valid data points due to insertion and recall biases, which are common in crowdsoursed data.

## Preprocessing and validation procedures performed by the original authors

Kaplanis et al. (2018) provide an in-depth description of the dataset, including a detailed explanation of the steps they performed to preprocess and enrich the dataset. Most of the details on the dataset can be found in the supplementary material accompanying the original paper. We briefly summarize the key steps of their process in the following.

After collecting the data from Geni.com, Kaplanis et al. removed invalid relationships, i.e., cycles and multi-parents, from the dataset. The authors focus on the largest strongly connected component of the family tree, a directed bipartite graph, which represents individuals, their unions (assumed to be marriages), and their relationships.

### Cycles

First, the authors removed relationships resembling cycles (relationships identifying children as parents of their ancestors). The removal of cycles had a minimal effect on the topology of the family tree and reduced the size of the largest connected component by 0.3% (to 15.3 million individuals).

### Multi-parents

Second, the authors identified and resolved multi-parent relationships, i.e., constellations, in which more than two parents were assigned to a child. The process involved several steps required to identify and resolve such relationships. The authors present an algorithm that identifies multi-parent relationships and resolve them by removing such events from the dataset or by locally merging duplicate entries responsible for the invalid relationship. To validate the merging algorithm applied during the clean-up process, the authors evaluated its accuracy based on a random sample of 1000 merged profiles, which were analyzed by experts. Compared to the experts' performance in merging duplicate profiles involved in multi-parent relationships, the authors' algorithm achieved an estimated accuracy of 94%. After applying the algorithm to the dataset, the authors removed 15% of the individuals in the largest connected component, resulting in a set of 13 million users.

### Non-paternity rate

Kaplanis et al. further validate the dataset based on data on genetic markers, which they obtained for a small subset of the actors comprising the dataset. They find that the non-paternity rate in the dataset is comparable to the non-paternity rate in Europeans, which has been established in previous studies, including a comprehensive meta-analysis.

### Annotation of geographic data

User-approved annotations: Geographic data is part of the original dataset. Geni.com users can add the birth location, current residence, death location, and burial location to a recorded person via a visual interface, which features a map to pinpoint locations and a form to provide textual information on the respective locations. The text-field is linked to a location database. In the case of a matching location, the full location record for the corresponding location is added to the respective profile.

Automated geo-parsing: Since not all location records are complete, Kaplanis et al. used a Yahoo service to those complete location records, which provide a sufficient amount of information to successfully perform a reliable lookup.

The authors compare the automated lookup to manually curated locations to validate their method. They do not find major deviations between both approaches and conclude that their lookup is valid.

### Further validations

Kaplanis et al. perform further validations on key demographic parameters, including a technical validation of the age of death and a comparison of Geni to traditional geographic data.

## Key variables

### Age at death

We compute the age at death by subtracting an actor's death date from the birthdate. For the first sample, in which valid records are only required to have a valid birth and death year, we compute the approximate age at death based on the difference in years. For the second sample, in which we require the full death and birthdates to be present, we compute the exact difference in dates to derive the precise age at death.

### Offspring-related metrics

For each actor in the dataset, we compute the number of offspring (m/f/total), the gender of the first and last offspring, and the age at birth of the first and last offspring (m/f/total). We compute the variables based on a Python script that iterates over every record in the actors' table of the dataset. For each individual included in the dataset, we extract all of their offspring from the relations table. For each offspring identified in the process, we check if their unique profile ID is present in the dataset. If it is present, we extract their gender and birthdate. Based on the birthdate, we identify the age at birth of the parent for each offspring. Based on this information, we define the age at first birth of the parent as the minimum absolute difference in years between the parent's and each offspring's birthdate (m/f/total). Similarly, we define the age at last birth (m/f/total) to be the maximum absolute difference between the parent's and each offspring's birthdate. The number of children is defined as the total number of a parent's offspring identified during the process.
