# Supplementary material for: Examining the trade-offs between human fertility and longevity over three centuries using crowdsourced genealogy data
Source: PLoS One. 2021 Aug 5;16(8):e0255528. doi: 10.1371/journal.pone.0255528 (PMC8341544; doi:10.1371/journal.pone.0255528)
Supplement: S1 Table — (DOCX) [file pone.0255528.s002.docx]

**S1 Table. Sample cases by region, country, and birth cohorts**

| **Birth cohorts** | **Western Europe** | | | | | **Scandinavia** | | | | |
| --- | --- | --- | --- | --- | --- | --- | --- | --- | --- | --- |
|  | BEL | FRA | GBR | NLD | **Total** | DNK | FIN | NOR | SWE | **Total** |
| 1601-1625 | 129 | 670 | 945 | 37 | **1781** | 83 | 24 | 54 | 119 | **280** |
| 1626-1650 | 277 | 1797 | 653 | 61 | **2788** | 95 | 43 | 69 | 194 | **401** |
| 1651-1675 | 370 | 1497 | 477 | 66 | **2410** | 147 | 129 | 110 | 435 | **821** |
| 1676-1700 | 486 | 1695 | 581 | 114 | **2876** | 213 | 532 | 216 | 1312 | **2273** |
| 1701-1725 | 755 | 2046 | 739 | 219 | **3759** | 307 | 997 | 364 | 2728 | **4396** |
| 1726-1750 | 902 | 2201 | 929 | 531 | **4563** | 508 | 1877 | 589 | 3578 | **6552** |
| 1751-1775 | 1241 | 2351 | 1374 | 1516 | **6482** | 751 | 2314 | 910 | 4108 | **8083** |
| 1776-1800 | 1266 | 2606 | 2273 | 2289 | **8434** | 921 | 2192 | 1332 | 3886 | **8331** |
| 1801-1825 | 1293 | 2114 | 3476 | 3106 | **9989** | 1305 | 1951 | 2373 | 3931 | **9560** |
| 1826-1850 | 1059 | 1464 | 3860 | 2877 | **9260** | 1348 | 1792 | 3566 | 3924 | **10630** |
| 1851-1875 | 1102 | 1190 | 2806 | 2351 | **7449** | 1318 | 1774 | 3927 | 3787 | **10806** |
| 1876-1900 | 856 | 1062 | 2006 | 1213 | **5137** | 1135 | 1332 | 3776 | 2920 | **9163** |
| 1901-1910 | 329 | 404 | 654 | 402 | **1789** | 464 | 340 | 1128 | 781 | **2713** |
| **Total** | 10065 | 21097 | 20773 | 14782 | **66717** | 8595 | 15297 | 18414 | 31703 | **74009** |
| **Birth Cohorts** | **Central Europe** | | | | | | **Southern Europe** | | | |
|  | DEU | AUT | CZE | POL | CHE | **Total** | ESP | PRT | ITA | **Total** |
| 1601-1625 | 431 | 6 | 3 | 8 | 91 | **539** | 162 | 5 | 17 | **184** |
| 1626-1650 | 584 | 9 | 9 | 7 | 142 | **751** | 207 | 21 | 23 | **251** |
| 1651-1675 | 1249 | 15 | 17 | 7 | 171 | **1459** | 215 | 23 | 20 | **258** |
| 1676-1700 | 1598 | 19 | 28 | 26 | 153 | **1824** | 188 | 64 | 39 | **291** |
| 1701-1725 | 2282 | 22 | 26 | 42 | 185 | **2557** | 216 | 119 | 46 | **381** |
| 1726-1750 | 2362 | 31 | 27 | 323 | 145 | **2888** | 226 | 113 | 78 | **417** |
| 1751-1775 | 2681 | 58 | 40 | 437 | 105 | **3321** | 174 | 78 | 71 | **323** |
| 1776-1800 | 2862 | 252 | 80 | 605 | 107 | **3906** | 157 | 56 | 82 | **295** |
| 1801-1825 | 3638 | 420 | 215 | 715 | 134 | **5122** | 166 | 79 | 141 | **386** |
| 1826-1850 | 4031 | 458 | 464 | 823 | 176 | **5952** | 135 | 68 | 210 | **413** |
| 1851-1875 | 3133 | 748 | 429 | 880 | 141 | **5331** | 171 | 93 | 350 | **614** |
| 1876-1900 | 1936 | 595 | 136 | 1354 | 117 | **4138** | 271 | 116 | 871 | **1258** |
| 1901-1910 | 576 | 177 | 58 | 621 | 59 | **1491** | 139 | 61 | 293 | **493** |
| **Total** | 27363 | 2810 | 1532 | 5848 | 1726 | **39279** | 2427 | 896 | 2241 | **5564** |
