# Supplementary material for: Examining the trade-offs between human fertility and longevity over three centuries using crowdsourced genealogy data
Source: PLoS One. 2021 Aug 5;16(8):e0255528. doi: 10.1371/journal.pone.0255528 (PMC8341544; doi:10.1371/journal.pone.0255528)
Supplement: S2 Table — (DOCX) [file pone.0255528.s003.docx]

**S2 Table. Sensitivity analyses of the effect of an additional child on maternal lifespan (in years)**

|  | Model S1 | | |  | Model S2 | | |  | Model S3 | | |
| --- | --- | --- | --- | --- | --- | --- | --- | --- | --- | --- | --- |
|  | Multilevel Tobit model,  2+ children sample | | |  | Multilevel OLS,  50+ age sample | | |  | Multilevel OLS,  full sample | | |
|  | β | std. err. | p |  | β | std. err. | p |  | β | std. err. | p |
| N of children | -0.086 | 0.031 | 0.006 |  | -0.031 | 0.022 | 0.188 |  | -0.567 | 0.058 | <0.001 |
| Age at last birth | 0.492 | 0.015 | <0.001 |  | 0.058 | 0.013 | <0.001 |  | 0.499 | 0.040 | <0.001 |
| MBI | -0.135 | 0.078 | 0.085 |  |  |  |  |  |  |  |  |
| MBI^2^ | 0.008 | 0.004 | 0.069 |  |  |  |  |  |  |  |  |
| Kid losses |  |  |  |  |  |  |  |  | -2.467 | 0.319 | <0.001 |
| Region fixed effects | Yes | | |  | Yes | | |  | Yes | | |
| Cohort fixed effects | Yes | | |  | Yes | | |  | Yes | | |
| N (Total) | 50506 | | |  | 66238 | | |  | 81927 | | |
| N (Uncensored) | 41535 | | |  | NA | | |  | NA | | |
| N (Censored) | 8971 | | |  | NA | | |  | NA | | |

Note: Modeling results are used for sensitivity analyses comparing to the benchmark model in Table 2 (Model 3, Table 2). Model S1 uses the multilevel Tobit regression and further controls for the mean inter-birth interval (MBI) and its squared term for a female subsample with at least two children. Model S2 uses the multilevel OLS regression for a female subsample who died after age 50. Model S3 uses the multilevel OLS regression for the full female sample, yet further conditioning on the hypothetical “kid losses” to reimburse early-death women’s potential fertility. The metric variable “kid losses” for a woman who died before age 50 is calculated as the difference between her number of children observed and the complete female fertility rate by age 50 for each cohort-region subgroup. For those who lived through reproductive years, the hypothetical “kid losses” are set to zero.
