# Supplementary material for: Examining the trade-offs between human fertility and longevity over three centuries using crowdsourced genealogy data
Source: PLoS One. 2021 Aug 5;16(8):e0255528. doi: 10.1371/journal.pone.0255528 (PMC8341544; doi:10.1371/journal.pone.0255528)
Supplement: S3 Table — (DOCX) [file pone.0255528.s004.docx]

**S3 Table. Sensitivity analyses of the effect of an additional child on paternal lifespan (in years)**

|  | Model S1 | | |  | Model S2 | | |  | Model S3 | | |
| --- | --- | --- | --- | --- | --- | --- | --- | --- | --- | --- | --- |
|  | Multilevel Tobit model,  2+ children sample | | |  | Multilevel OLS,  50+ age sample | | |  | Multilevel OLS,  full sample | | |
|  | β | std. err. | p |  | β | std. err. | p |  | β | std. err. | p |
| N of children | 0.110 | 0.022 | <0.001 |  | -0.009 | 0.023 | 0.716 |  | -0.366 | 0.035 | <0.001 |
| Age at last birth | 0.349 | 0.008 | <0.001 |  | 0.063 | 0.008 | <0.001 |  | 0.333 | 0.017 | <0.001 |
| MBI | -0.037 | 0.051 | 0.474 |  |  |  |  |  |  |  |  |
| MBI^2^ | 0.002 | 0.002 | 0.394 |  |  |  |  |  |  |  |  |
| Kid losses |  |  |  |  |  |  |  |  | -3.101 | 0.285 | <0.001 |
| Region fixed effects | Yes | | |  | Yes | | |  | Yes | | |
| Cohort fixed effects | Yes | | |  | Yes | | |  | Yes | | |
| N (Total) | 63099 | | |  | 87672 | | |  | 103642 | | |
| N (Uncensored) | 54193 | | |  | NA | | |  | NA | | |
| N (Censored) | 8906 | | |  | NA | | |  | NA | | |

Note: Modeling results are used for sensitivity analyses comparing to the benchmark model in Table 3 (Model 3, Table 3). Model S1 uses the multilevel Tobit regression and further controls for the mean inter-birth interval (MBI) and its squared term for a male subsample with at least two children. Model S2 uses the multilevel OLS regression for a male subsample who died after age 50. Model S3 uses the multilevel OLS regression for the full male sample, yet further conditioning on the hypothetical “kid losses” to reimburse early-death men’s potential fertility. The metric variable “kid losses” for a man who died before age 50 is calculated as the difference between his number of children observed and the complete male fertility rate by age 50 for each cohort-region subgroup. For those who lived through reproductive years, the hypothetical “kid losses” are set to zero.
