# Supplementary material for: Examining the trade-offs between human fertility and longevity over three centuries using crowdsourced genealogy data
Source: PLoS One. 2021 Aug 5;16(8):e0255528. doi: 10.1371/journal.pone.0255528 (PMC8341544; doi:10.1371/journal.pone.0255528)
Supplement: S1 Fig — (DOCX) [file pone.0255528.s005.docx]

**S1 Fig. Comparing cohort fertility between subsamples with pre-menopausal and post-menopausal death.**

**
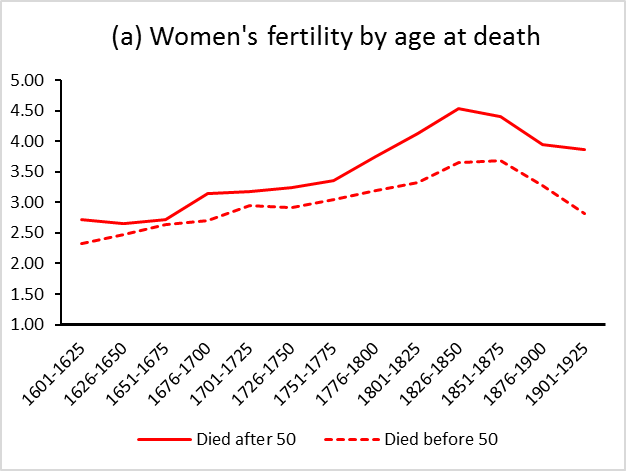
**


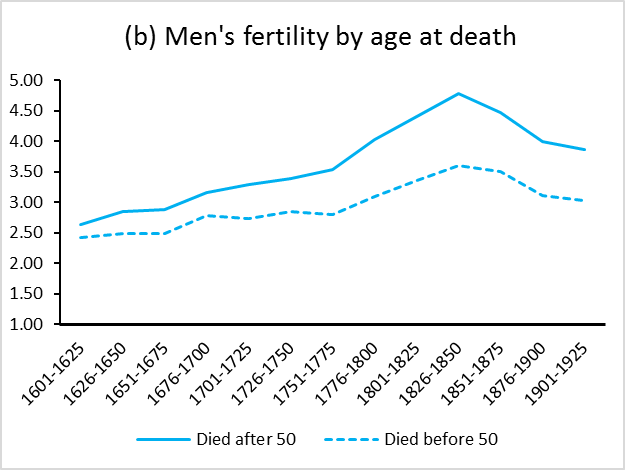


Source: Authors’ calculation from the FamiLinx dataset.
