# Supplementary material for: Examining the trade-offs between human fertility and longevity over three centuries using crowdsourced genealogy data
Source: PLoS One. 2021 Aug 5;16(8):e0255528. doi: 10.1371/journal.pone.0255528 (PMC8341544; doi:10.1371/journal.pone.0255528)
Supplement: S2 Fig — (DOCX) [file pone.0255528.s006.docx]

**S2 Fig. Crude birth rate (CBR) in England and cohort fertility rate (CFR) in our FamiLinx sample**

**
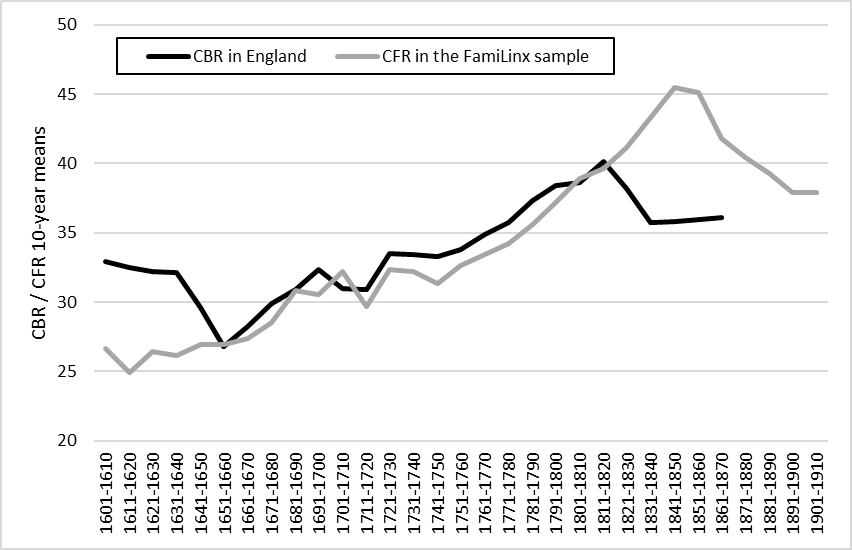
**

Source: The black-line CBRs in England are calculated based on Wrigley and Schofield (1980, Table A3) [59]; The gray-line CFRs are calcualated based on the FamiLinx dataset.

Note: For a rough comparison, demographic trends in cohort fertility indices generally lag about 30 years behind the period (hypothetical) fertility indices. In S2 Fig, the period CBR in England has increased from 26.8 in 1651–1660 and peaks at 40.1 during the years 1811–1820, indicating that the similar fertility trend and climax could be observed in a 30-year lagged pattern in the cohort CFR. Indeed, this pattern is what we found in our FamiLinx sample, where the CFR of the 1681-1690 cohorts is 30.8 and the climax of it is 45.5 children per thousand women for the 1841–1850 cohorts. The five-child gap between the CBR in England and our CFR might result from the differences between period-based and cohort-based fertility calculation: in pre-industrial scenario where infant and youth mortality was high, the period fertility indices tend to record a much lower fertility rate comparing to the cohort fertility indices.
