# Supplementary material for: Examining the trade-offs between human fertility and longevity over three centuries using crowdsourced genealogy data
Source: PLoS One. 2021 Aug 5;16(8):e0255528. doi: 10.1371/journal.pone.0255528 (PMC8341544; doi:10.1371/journal.pone.0255528)
Supplement: S3 Fig — (DOCX) [file pone.0255528.s007.docx]

**S3 Fig. Effect of an additional child on lifespan (in years) by birth cohorts, results from different models and samples.**


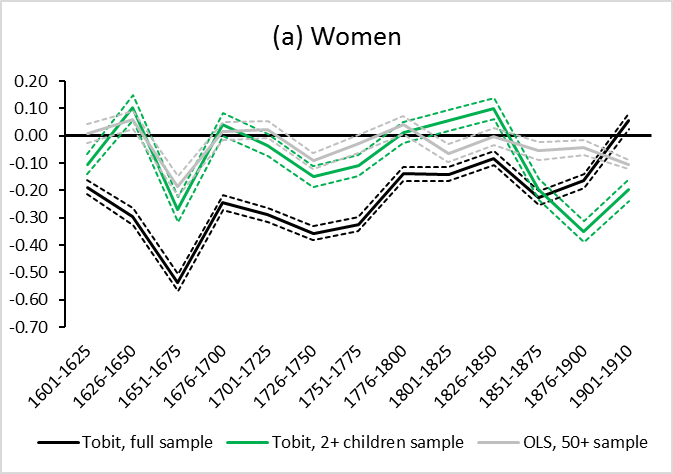


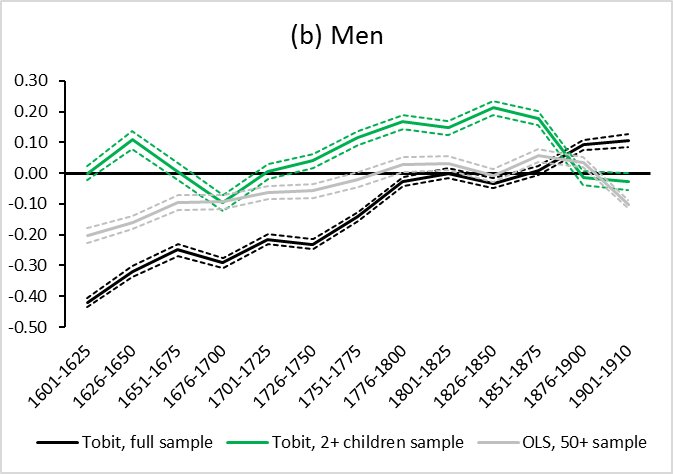


Note: Solid lines present the point estimates of fertility effects; dashed lines present the 95% confidence intervals. Black lines present the estimation results from our focal models using the full sample and multilevel Tobit regressions (same as Fig. 2 in the main text). Green lines present the results from the first sensitivity analysis using the 2+ children subsample and multilevel Tobit regressions, conditioning on MBI (S2 Table, Model 1 for women; S3 Table, Model 1 for men). Gray lines present the results from the second sensitivity analysis using the 50+ subsample and multilevel OLS regressions (S2 Table, Model 2 for women; S3 Table, Model 2 for men).

Firstly, Model (b) might outperform Model (a) during a historical period of high natural fertility (i.e., reduce the sample selection bias) and low risk of birth-related death (i.e., reduce the overadjustment bias). In Europe, this roughly refers to an early-industrial period from the mid- to the late-nineteenth century before introducing the population-level contraception [58]. Secondly, Model (c) might outperform Model (a) during a historical period of longer lifespan when survival beyond 50 years old is common regardless of health and socioeconomic status, which also refers to the period after the mid-nineteenth century. Finally, we can use the estimation results from Model (c) to check for the risk of reverse causality in Model (a). That is, it might be one’s lifespan that determines her/his fertility rather than vise versa. Under the assumption that a longer lifespan positively associates with higher fertility, we might expect an even stronger negative effect of fertility on longevity in Model (c) comparing to Model (a) if the reverse causality is dominating. This is because Model (c) has largely ruled out the risk of reverse causality by focusing only on the post-reproductive subsample, thereby exempting from the positive association between fertility and lifespan. On the other hand, if a stronger negative effect in Model (a) comparing to Model (c) is observed, we might expect that the negative causal effect of fertility on longevity is dominating even among those with earlier death.
